# Determinants of time-to-disposition in patients who underwent CT for pulmonary embolism: a retrospective study

**DOI:** 10.1186/s12873-021-00510-7

**Published:** 2021-10-12

**Authors:** Ali Hassan, Omran Al Dandan, Khaled Awary, Besma Bukhamsin, Reema Bukhamseen, Alaa Alzaki, Amal Al-Sulaibeekh, Hind S. Alsaif

**Affiliations:** 1 Department of Radiology, Salmaniya Medical Complex, Manama, Bahrain; 2 Department of Radiology, King Fahd Hospital of the University, Imam Abdulrahman Bin Faisal University, Al-Khobar, Saudi Arabia; 3 Department of Internal Medicine, King Fahd Hospital of the University, Imam Abdulrahman Bin Faisal University, Al-Khobar, Saudi Arabia; 4 Department of Emergency Medicine, King Fahd Hospital of the University, Imam Abdulrahman Bin Faisal University, Al-Khobar, Saudi Arabia

**Keywords:** Pulmonary embolism, Emergency department, Length of stay, 4-h target

## Abstract

**Background:**

Pulmonary embolism (PE) is a common life-threatening medical emergency that needs prompt diagnosis and management. Providing urgent care is a key determinant of quality in the emergency department (ED) and time-based targets have been implemented to reduce length of stay and overcrowding. The study aimed to determine factors that are associated with having a time-to-disposition of less than 4 h in patients with suspected PE who underwent computed tomography pulmonary angiography (CT-PA) to confirm the diagnosis.

**Methods:**

After obtaining approval from the ethics committee, we conducted a retrospective observational study by examining CT-PA scans that was performed to rule out PE in all adult patients presenting at the ED between January 2018 and December 2019. Demographic information and clinical information, as well as arrival and disposition times were collected from electronic health records. Multivariable regression analysis was used to identify the independent factors associated with meeting the 4-h target in the ED.

**Results:**

In total, the study involved 232 patients (76 men and 156 women). The median length of stay in the ED was 5.2 h and the 4-h target was achieved in 37% of patients. Multivariable logistic regression analysis revealed that a positive CT-PA scan for PE was independently associated with meeting the four-hour target in the ED (odds ratio [OR]: 2.2; 95% *CI*: 1.1–4.8). Furthermore, Hemoptysis was the only clinical symptom that served as an independent factor associated with meeting the 4-h target in the ED (*OR*: 10.4; 95% *CI*: 1.2–90.8).

**Conclusion:**

Despite the lower number of staff and higher volume of patients on weekends, patients who presented on weekends had shorter stays and were more likely to meet the 4-h target. Careful clinical assessment, prior to requesting a CT-PA scan, is crucial, since negative CT-PA scans may be associated with failure to meet the 4-h target.

## Background

Pulmonary Embolism (PE) is a common life-threatening medical condition that is often misdiagnosed since it lacks characteristic clinical features. Computed tomography pulmonary angiography (CT-PA) has become the first-line imaging modality of choice for the diagnosis of PE because of its high accuracy and non-invasive nature. The mortality from untreated PE can reach up to 30%. Notably, it is estimated that two thirds of mortalities occur within the first 2 h after clinical presentation [1]. Hence, timely diagnosis and management is essential due to the substantial effect on mortality and morbidity [2].

Providing urgent care is a key focus in the emergency departments (ED) as delays may lead to significant adverse outcomes [3]. Reducing the length of stay in the ED has thus attracted much attention as a key determinant of quality [4, 5]. Overcrowding in EDs has been associated with delays in the administration of medications, admission to the wrong wards, and increased medical errors [6]. Crowding could have negative effects on healthcare providers, including increased risk of violence and decreased job satisfaction [7, 8].

In our institution, the 4-h target, which aims to limit the time between arrival and disposition decision to a maximum of 4 h, was introduced in 2016 resulting in a decreased length of stay in the ED. This time-based target was first implemented by the National Health Service (NHS) in the United Kingdom in 2004 and was adopted by the Australian government in 2012 [9, 10]. However, the NHS has modified its target to 95% of patients to allow for certain clinical exceptions [11]. Several studies have demonstrated the benefits of the 4-h rule. Waiting times in the ED were significantly reduced and a reduction in mortality rates following the introduction of time-based targets has also been demonstrated [9, 12, 13].

While the use of complex imaging is often thought to be a culprit of prolonged ED stays [11], it seems unlikely to be a causative factor for breaching time-based targets. For example, a retrospective study revealed that most patients who exceeded the target had stayed over 4 h after the imaging itself was completed [14]. Several studies have explored the determinants of length of stay in EDs [11, 15, 16]. However, it would be of special interest to investigate the length of stays for the group of patients with suspected PE who underwent imaging to rule it out.

Considering the widespread availability of CT-PA, it is unsurprising to see a low threshold for its use in the diagnosis of PE [17]. It remains unclear, however, whether CT-PA results could influence the length of stay in the ED. Time-based targets could potentially compel physicians not to request a D-dimer assay prior to a CT-PA scan even if the patient is deemed unlikely to have PE [18]. No previous study has investigated the effects of skipping the D-Dimer assay and proceeding directly to a CT-PA scan on compliance with time-based targets. Furthermore, radiology report turnaround times could be higher during on-call hours where a consultant radiologist may not be immediately available [19]. This could influence compliance with time-based targets in the ED according to the shift periods.

Therefore, this study aimed to investigate the length of stay among patients who underwent CT-PA scans for suspected PE and to determine the patient and environmental factors that serve as independent factors associated with meeting the 4-h target in ED.

## Materials and methods

### Study design

After obtaining approval from the ethics committee, we conducted a retrospective study to investigate the determinants of length of stay for patients who underwent CT-PA for suspected PE and the compliance with the 4-h target in the ED.

### Study setting

The study was conducted at the King Fahd Hospital of Imam Abdulrahman Bin Faisal University, an academic center located in the Eastern Province of Saudi Arabia. Each year, approximately 250,000 patients visit the ED. CT-PA scans can only be requested by board-certified consultant physicians.

### Study population

The Radiology Information System was used to identify all requests for CT-PA scans for patients presenting at the ED between January 2018 and December 2019. Eligible participants were adult patients who underwent CT-PA to rule out PE. Exclusion criteria were as follows: age below 18 years, pregnancy, and the purpose of the scan was not to rule out PE.

### Data collection

A structured data collection form was created using the QuestionPro platform (Seattle, WA, USA) to collect data from patient electronic health records that included:

- *Patient-Related Information*: Age, sex, co-morbidities, presenting sign and symptoms, D-dimer level, and CT-PA findings were obtained. The Charlson Comorbidity Index (CCI) score, an important measure of burden of disease [20], was calculated for each patient. Tachycardia was defined as heart rate > 100 bpm, hypotension was defined as blood pressure < 90/60 mmHg, and hypoxia was defined as an oxygen saturation < 95% on room air. The Wells score was calculated retrospectively from the data available in the electronic health record in case the score was not explicitly stated in the patient record.
- *Environment-Related Information*: Data on date and time of presentation and disposition were obtained. Length of stay was estimated as the difference between the time of presentation and the time of disposition (discharge, admission, or referral).

The data collection process was performed by a diagnostic radiology resident who was familiar with the Hospital Information System and received adequate training and supervision by the principal investigator. The data was checked by the principal investigator and inconsistent data was revised by referring to the electronic records again.

### Statistical analysis

After checking for completeness and accuracy, data were analyzed using IBM SPSS for Windows, version 26 (IBM Corp., Armonk, NY, USA). Categorical variables, presented as percentages and frequency distribution, were compared using the chi-squared or Fisher’s exact tests, as appropriate. Continuous variables, presented as median and interquartile range (IQR), were compared using the Mann-Whitney U and Kruskal-Wallis tests, as appropriate. The Wells score was dichotomized according to the two-tier model with the “PE likely” group has a score > 4.0 and the “PE unlikely” group has a score ≤ 4.0. Multivariable binary logistic regression analysis was conducted to identify the independent factors associated with meeting the 4-h target in the ED. Candidate variables were selected based on risk factors identified in the literature and bivariate analyses. Odds ratio (OR) with 95% confidence interval (CI) were estimated using the full model fit and were reported in comparison with the designated reference group. The goodness-of-fit of the model was evaluated using the Omnibus and Hosmer-Lemeshow tests. The significance level was defined as α = 0.05. The Bonferroni correction was used for multiple comparisons.

## Results

### Patient characteristics

The study involved 232 patients (76 men and 156 women) who presented to the ED and underwent CT-PA scan to rule out PE. Only 34 (14.6%) patients had a PE and a D-dimer assay was requested for 137 (59.1%) patients. Table 1 summarizes the information pertaining to the clinical encounters according to patient and environmental factors.

**Table 1:** Characteristics of clinical encounters according to patient and environmental factors

Variables			N (%)	
Patient Factors	Age < 50 years		186	(80.2)
	Male Sex		76	(32.8)
	Charlson Comorbidity Index	0	110	(47.6)
		1–2	57	(24.6)
		3–4	28	(12.1)
		≥ 5	37	(15.9)
	Clinical Symptoms	Asymptomatic	9	(3.9)
		Dyspnea	176	(75.9)
		Chest Pain	124	(53.4)
		Cough	57	(24.6)
		Hemoptysis	7	(3.0)
		Leg Pain or Swelling	23	(9.9)
		Syncope	7	(3.0)
	Clinical Signs	Tachycardia	129	(55.6)
		Hypoxia	59	(25.4)
		Hypotension	6	(2.6)
		Altered Consciousness	14	(6.0)
	Ordered D-Dimer Assay		95	(40.9)
	Positive CT-PA for PE		34	(14.7)
	High Probability of PE based on Wells Criteria		50	(21.6)
Environmental Factors	Year of Presentation	2018	136	(58.6)
		2019	96	(41.4)
	Presented in October (Beginning of Academic Year)		176	(75.9)
	Presented in Weekend		56	(24.1)
	Shift Period	Morning	65	(28.0)
		Evening	97	(41.8)
		Night	70	(30.2)

*Abbreviation*: *N* Number of encounters, *CT-PA* Computed tomography pulmonary angiography, *PE* Pulmonary embolism, *ED* Emergency department

### Time-to-disposition

The median time-to-disposition from the ED was 5.2 h (*IQR*: 2.8–7.0). The time-to-disposition according to different patient and environmental factors is summarized in Table 2. On bivariate analysis, there was no significant difference in the time-to-disposition according to the patient’s age, sex, CCI, or presenting symptoms. However, the time-to-disposition was significantly lower in patients who presented with hypoxia (4.2 h vs. 5.3 h; *U* = 4171.0, *P* = 0.04) and altered level of consciousness (3.1 h vs. 5.3 h; *U* = 887.5, *P* = 0.01). The time-to-disposition of patients who had a D-dimer assay was longer than that of patients who did not have the assay (5.3 h vs. 4.9 h), although the difference was not statistically significant (*U* = 6110.5, *P* = 0.43). The patients who were found to have a PE in the CT-PA had a significantly shorter length of stay (3.7 h vs. 5.3 h; *U* = 2497.5, *P* = 0.02).

**Table 2:** Time-to-disposition according to different patient and environmental factors

Variables			Median Time to Disposition (Hours)	*P* value ^a^	Meeting the Four-Hour Target (%)	*P* value ^a^
Patient Factors	Age	< 50 years	5.2	0.87	37	0.75
		≥ 50 years	5.4		39	
	Gender	Male	4.5	0.09	45	0.09
		Female	5.4		33	
	Charlson Comorbidity Index	0	4.8	0.32	40	1.00
		1–2	5.4		35	
		3–4	6.2		21	
		≥ 5	4.5		43	
	Clinical Symptoms	Asymptomatic	6.8	0.34	22	0.35
		Dyspnea	5.0	0.29	39	0.23
		Chest Pain	5.0	0.45	40	0.41
		Cough	5.2	0.46	37	0.97
		Hemoptysis	3.1	0.11	86	**0.01**
		Leg Pain or Swelling	4.7	0.38	48	0.26
		Syncope	5.6	0.82	29	0.64
	Clinical Signs	Tachycardia	4.9	0.69	39	0.55
		Hypoxia	4.2	**0.04**	46	0.11
		Hypotension	4.4	0.34	17	0.29
		Altered Consciousness	3.1	**0.01**	64	**0.03**
	D-Dimer Assay	Ordered	5.3	0.43	34	0.37
		Not Ordered	4.9		39	
	CT-PA Report	Positive for PE	3.8	**0.02**	53	**0.04**
		Negative for PE	5.3		34	
Environmental Factors	Year of Presentation	2018	5.3	0.86	38	0.87
		2019	5.2		36	
	Month of Presentation	October (Beginning of Academic Year)	5.5	0.88	37	0.62
		Other Months	5.2		44	
	Day of Presentation	Weekday	5.6	**0.01**	34	**0.047**
		Weekend	4.1		48	
	Shift Period	Morning	4.6	1.00	42	1.00
		Evening	5.2		37	
		Night	5.5		33	

*Abbreviation*: *N* number of encounters, *CT-PA* Computed tomography pulmonary angiography, *PE* Pulmonary embolism, *ED* Emergency department

^a^ *P* value in bold when significant

There was a significant difference in the time-to-disposition in patients according to the day of presentation. Patients who presented over the weekend had a shorter length of stay by approximately half an hour when compared with patients presenting on weekdays (4.1 h vs. 5.6 h; *U* = 3845.5, *P* = 0.01). However, there was no significant difference according to the year or month of presentation or the shift period (*P* > 0.05).

### Compliance with the 4-hour target

The 4-h target was achieved in 37% of patients. As shown in Table 2, the compliance rate did not significantly differ according to patient demographic factors. Overall, 86% of patients presenting with hemoptysis stayed in the ED for less than 4 h compared to 36% of patients who did not present with hemoptysis (χ^2^ = 7.322, *P* = 0.01). Similarly, 64% of patients presenting with altered consciousness met the 4-h target compared to 35% of patients with a normal level of consciousness (χ^2^ = 4.731, *P* = 0.03).

### Multivariable analysis of factors associated with meeting the 4-hour target

Multivariable binary logistic regression analysis was conducted to identify patient and environmental factors that are associated with meeting the 4-h target in the ED after controlling for the likelihood of PE based on the Wells criteria. A test of the full model vs. the intercept-only model was statistically significant (χ^2^ = 21.1, *P* = 0.004).

The model revealed that the CT-PA result was an independent factor associated with meeting the 4-h target, as patients who were found to have PE had a higher tendency of staying less than 4-h in the ED (*OR*: 2.2; 95% *CI*: 1.1–4.8). Hemoptysis was the only clinical symptom that served as an independent factor associated with meeting the 4-h target in the ED (*OR*: 10.4; 95% *CI*: 1.2–90.8) (Table 3).

**Table 3:** Multivariable logistic regression of factors associated with meeting the four-hour target

Variables	Univariable Logistic Regression			Multivariable Logistic Regression		
	OR	[95% CI]	*P* value ^a^	OR	[95% CI]	*P* value ^a^
Age ≥ 50 years	0.9	[0.5, 1.7]	0.75	1.0	[0.5, 2.1]	0.95
Male patient	0.6	[0.4, 1.1]	0.09	1.4	[0.8, 2.5]	0.29
Hemoptysis	10.9	[1.3, 91.9]	**0.03**	10.4	[1.2, 90.8]	**0.03**
Altered consciousness	3.3	[1.1, 10.2]	**0.04**	3.0	[0.9, 10.0]	0.08
CT-PA was positive for PE	2.2	[1.03, 4.5]	**0.04**	2.2	[1.1, 4.8]	**0.02**
Weekend presentation	1.8	[1.0, 3.4]	**0.049**	2.0	[0.9, 3.3]	0.10
“PE likely” Wells score	1.6	[0.9, 3.0]	0.14	1.2	[0.6, 2.4]	0.57

*Abbreviation: CI* Confidence interval, *CT-PA* Computed tomography pulmonary angiography, *OR* Odds ratio, *PE* Pulmonary embolism

^a^ *P* value in bold when significant

## Discussion

Our study aimed to investigate the factors that determined the length of stay and compliance with the 4-h target in the ED for patients who underwent CT-PA to rule out PE. We found that a positive CT-PA for PE and hemoptysis were independent factors associated with meeting the 4-h target in the ED.

A positive CT-PA scan was found to be associated with a shorter length of stay in the ED, suggesting that unjustified CT-PA scans may delay disposition and discharge of patients with suspected PE. This finding may suggest that having a clear diagnosis could make disposition easier and faster. While there is no clinical protocol in place for PE, it is the usual practice in our institution to refer patients with confirmed PE to the hematology team for further management. Several studies have demonstrated the value of having clear clinical protocols in the ED for the patient care, including reducing length of stays [21, 22]. Furthermore, incidental findings in CT-PA scans are common [23], and previous studies have demonstrated that incidental radiological findings were associated with longer stays [24, 25].

Although there is no accurate data about variation in the use of EDs according to day of the week, anecdotal information suggests that use is higher on the weekends. Moreover, a retrospective study conducted in the United States showed that EDs were visited more often on weekends [26]. It was demonstrated in previous research that the root causes of longer stays in the ED were mostly organizational in nature and beyond the control of the ED [27].

Counterintuitively, the study found that these weekend ED visits had shorter stays and higher compliance with the 4-h rule. A retrospective single-institution study involving more than 300,000 ED visits had a similar pattern revealing that the highest use of the ED on weekends, but the highest probability of breaching the time-based targets was on days following weekends [11]. This interesting finding was attributed to the high number of admissions on weekends resulting in a shortage of inpatient beds on the weekdays and delays in admitting the patients to inpatient beds [11, 28]. While this explanation is reasonable, we also assume that because outpatient clinics are not available on the weekends, the turnaround time for radiological and laboratory tests for patients presenting at the ED could be shorter, which facilitating the diagnosis and disposition of patients on weekends. However, the multivariable model did not show a significant difference in achieving the 4-h target based on the day of presentation to the ED. It is worth-mentioning that there was not a significant difference in length of stay or compliance with the 4-h target in October, which is the beginning of the academic year in our institution, compared to other months.

Radiology departments may be affected by time-based targets in ED. A retrospective study by Tse et al. [14] to assess trends in requests for radiology, before and after introduction of a 4-h standard, showed an increase of 60% after introduction of the rule. The study also revealed a shift toward the use of more CT scans compared with plain X-ray images [14]. Current guidelines for the management of PE recommend using the D-dimer assay in patients with a low pre-test probability of PE [29]. A recent study from our institution revealed that one-third of all CT-PA scans requested by physicians in the ED did not adhere to clinical decision rules and that the D-dimer assay was significantly underused. Therefore, it has been suggested that time targets in EDs may compel physicians to request a CT-PA scan without having a D-dimer assay, even if the patient is deemed unlikely to have PE [30]. While the present study found that there was a notable difference of approximately half an hour in the length of stay between patients who had D-dimer assay and those who did not, it should be noted that our study included the patients who underwent a CT-PA scan only. The predictive value of normal D-dimer levels for exclusion of PE was over 99% and clinical probability, combined with a D-dimer assay, may thus exclude the need for further investigations and could reduce the length of stay significantly [31]. In the appropriate setting, ED physicians should, therefore, not be reluctant to request a D-dimer assay just for the sake of meeting time-based targets.

Our study had certain limitations. It represented a single-center experience and included all patients with suspected PE who presented at the ED over 2 years. We also did not collect detailed descriptions of events during the ED stay, such as time from image request to discharge, which could have given a better understanding of contributions to length of stay.

## Conclusion

The meeting of the 4-h target was not significantly associated with most of the patient and environmental factors. Careful clinical assessment, prior to requesting a CT-PA scan, is crucial, because negative CT-PA scans may be associated with failure to meet the 4-h target. The findings of this study may enable stakeholders to implement appropriate interventional measures to reduce the probability of exceeding the 4-h target.

## Data Availability

The datasets used and/or analyzed during the current study are available from the corresponding author on reasonable request.

## Abbreviations

CI: Confidence interval
CCI: Charlson comorbidity index
CT-PA: Computed tomography pulmonary angiography
ED: Emergency department
OR: Odds ratio
PE: Pulmonary embolism
